# The electrophysiological tests in the early detection of the visual pathway dysfunction in patients with microadenoma

**DOI:** 10.1007/s10633-021-09827-x

**Published:** 2021-03-20

**Authors:** Ewelina Lachowicz, Wojciech Lubiński, Wojciech Gosławski, Elżbieta Andrysiak-Mamos, Agnieszka Kaźmierczyk-Puchalska, Anhelli Syrenicz

**Affiliations:** 1 II Department of Ophthalmology, Pomeranian Medical University, SPSK-2, Powstańców Wlkp. 72, 70-111 Szczecin, Poland; 2 Clinic of Endocrinology, Metabolic and Internal Diseases, Pomeranian Medical University, SPSK-1, Szczecin, Poland

**Keywords:** Pituitary tumour, Microadenoma, Visual pathway dysfunction, PVEP, MVEP, PERG

## Abstract

**Purpose:**

To evaluate the validity of electrophysiological tests in the early diagnosis of a ganglion cells and/or optic nerve dysfunction in patients with pituitary microadenoma.

**Methods:**

66 eyes, from 33 patients with microadenoma with no evidence of the optic chiasm compression in magnetic resonance imaging (MRI) and the visual impairment in the routine ophthalmological examination, standard static perimetry (24-2 white on white) and optical coherence tomography (HD-OCT), were analysed. The pattern electroretinogram (PERG), standard pattern visual evoked potentials (PVEPs) and multichannel visual evoked potentials (mVEPs) (ISCEV standards) were performed. The results obtained from the electrophysiological tests were compared to the same number of age-matched healthy controls.

**Results:**

Statistically significant differences between the patients with microadenoma and healthy controls were detected in all electrophysiological tests (*p* < 0.001). The most frequent abnormalities were observed in mVEPs (25/33 patients, 75.8%; 43/66 eyes, 65.2%). The most frequent features registered in this test were: (1°4′)—an increase in the P100wave latency from uncrossed fibres (13/33 patients, 39.39%; 21/66 eyes, 31.8%) and (0°16′)—an amplitude reduction of this wave from the crossed fibres (11/33 patients, 33.33%; 19/66 eyes, 28.8%). The changes in PVEPs (15/33 patients, 45.5%; 25/66 eyes, 37.9%) and PERG (10/33 patients, 30.3%; 15/66 eyes, 22.7%) were also registered. Of all the tests and parameters analysed in the study, the greatest diagnostic value in detecting the visual pathway dysfunction in this group of patients was the amplitude of P100 wave from the crossed fibres of the mVEPs (1°4′) with a sensitivity of 60.6% and a specificity of 93.8%. These parameters suggest that this type of dysfunction is downstream to the chiasm and can also indicate the visual pathway dysfunction severity.

**Conclusions:**

In patients with microadenoma, the abnormalities in the electrophysiological tests are registered even without clinical evidence of visual impairment from the routine ophthalmological examination, SAP, OCT and chiasmal compression in MRI. The mVEPs have the most significant role in the diagnosis of the visual pathway dysfunction in patients with microadenoma.

## Introduction

The main cause of chiasmal dysfunction is pituitary adenomas. It represents the most frequent intrasellar pathology and accounts for about 10–15% of all intracranial tumours [1, 2]. About 70% of pituitary adenomas occur in women aged 30–50 years [3]. Approximately 50% are microadenomas (dimension < 10 mm); the remainder are macroadenomas (dimension ≥ 10 mm). Functional adenomas occur in 70% of cases and prolactinoma are the most common secreting subtype, accounting for about 32–66% of these tumours [2–5]. The unique relationship of the optic chiasm to the pituitary gland can often lead to visual pathway impairment from the extension of the pituitary tumour, even in the early stage of the disease, and can cause the optic nerve damage. Visual dysfunction is the main presenting symptom. It is commonly known that microadenoma does not affect the optic chiasm. In opposition to this suggestion, the available literature describes a small number of cases with pituitary tumours where the visual acuity and can be normal, while significant ganglion cell and/or optic nerve deterioration can be observed [6–15]. The study results mentioned above indicate that, in patients with pituitary tumours even in the absence of compressive effect at the chiasm on MRI, the visual pathway dysfunction can be detected by electrophysiological tests.

That it is why we decided to conduct a study of large group of patients with microadenoma to elucidate the influence of small pituitary tumours on the visual pathway function.

## Patients and methods

Sixty-six eyes belonging to 33patients (27 females, 6 males) with a mean age of 33.7 ± 9.9 years with microadenoma detected in MRI were analysed. In all patients, the following examinations were performed: routine ophthalmological evaluation, the distance best corrected visual acuity (DBCVA, Snellen chart), tonometry (Pascal tonometer), anterior and posterior segment assessment (slit lamp, Volk lens), colour perception (the Farnsworth-Munsell Dichotomous D-15 test), as well as a retinal sensitivity measured by standard static perimetry (SITA 24-2 white on white threshold, Humphrey Visual Field Analyzer), the thickness of circumpapillary retinal nerve fibres layer (RNFL) and the ganglion cell complex (GCC) thickness estimated in optical coherence tomography (Cirrus HD-OCT 5000, Carl Zeiss Meditec, Dublin, CA, USA). The inclusion criteria were: pituitary tumour size < 10 mm confirmed by MRI, no ophthalmic symptoms, normal results from routine ophthalmological examination, perimetry and OCT. Furthermore, cooperative patients who agreed to participate in follow-up visit were enrolled in a study group. Patients with ocular (e.g. glaucoma, high refractive errors and others) or systemic diseases (e.g. unregulated diabetes mellitus, heart failure, depression and others), or who were taking medications(e.g. digoxin, metronidazole, oxazepam, quinolones, thioridazine and others) with known influence on the function of the retina and optic nerve were excluded.

In the selected group of patients, electrophysiological tests were conducted, as described previously [16]: pattern electroretinogram (PERG), pattern visual evoked potentials (PVEPs) and multichannel visual evoked potentials (mVEPs), according with International Society for Clinical Electrophysiology of Vision (ISCEV) Standards [17, 18]. The following parameters were analysed in the tests: PVEP—amplitude of P100 wave (AP100), latency of P100 wave (LP100); mVEP—amplitude of P100 wave (AP100), latency of P100 wave (LP100) from crossed and uncrossed fibres; PERG—amplitude of P50 wave (AP50), amplitude of N95 wave (AN95), peak time of P50 wave (PTP50).

It is worth knowing that the multi-channel VEPs recording is not required for the basic ISCEV standard of clinical VEP. However, assessment of the chiasmal and post-chiasmal visual pathway dysfunction requires multi-channel recording for proper diagnosis. Dysfunction of the chiasm gives a crossed asymmetry whereby the lateral asymmetry obtained on stimulation of one eye is reversed when the other eye is stimulated. The pattern stimuli should be presented with a field of 30 degree. A minimum of two channels are needed to detect lateral asymmetries. The minimum of the three active electrodes (two lateral electrodes placed at O1 and O2 and a third midline active electrode at Oz) referenced to Fz should be used. Particular caution is needed when interpreting multi-channel pattern-reversal VEPs because of paradoxical lateralization. This phenomenon, in which the response recorded at a lateral scalp location is generated by activity in the contralateral hemisphere of the brain, occurs with a large field, large check reversal stimulus and common reference recording to Fz [18]. That bipolar recordings using ipsilateral hemisphere reference electrodes do not show paradoxical lateralization of the signal recorded via Fz reference is thus apparent [19]. When the tumour is more posterior and lateral to one side of the optic chiasm, the VEPs can show an uncrossed, asymmetrical distribution [6–8, 20–22].

The results obtained from the electrophysiological tests were compared with the same number of age-matched healthy controls.

### Statistical analysis

The values of PVEP/mVEP/PERG parameters, from both the patients with a pituitary tumour and the control group, were statistically analysed. The assumption of normality was checked using the W Shapiro–Wilk test. In reference to the normality tests, the norm ranges have been determined based on the values of parameters from the control group. In the case of the normal distribution of the variable, the range of the normal limits was between ± 2SD. In the absence of normality, the range of the normal limits was between 2.5 and 97.5 percentile. The values of the analysed parameters of the electrophysiological tests between the two groups were compared. Depending on the variable distribution, two different tests were used; parametric or nonparametric, the Student’s t test or Mann–Whitney U test, respectively. The results were considered as a statistically significant with *p* < 0.05(*p < 0.05, **p < 0.01, ***p < 0.001).

PERG/VEP interocular/intercortical asymmetry was determined based on methodology described in literature [21, 23].

The sensitivity and specificity of each parameter of the electrophysiological examination were determined on the basis of the cut-off point on the ROC curve (receiver operating characteristic). In addition, the area under the ROC–AUC (area under curve) curve was calculated. Based on the AUC value, the predictive ability of the parameters in relation to the random model was also determined.

## Results

**Fig. 1:**
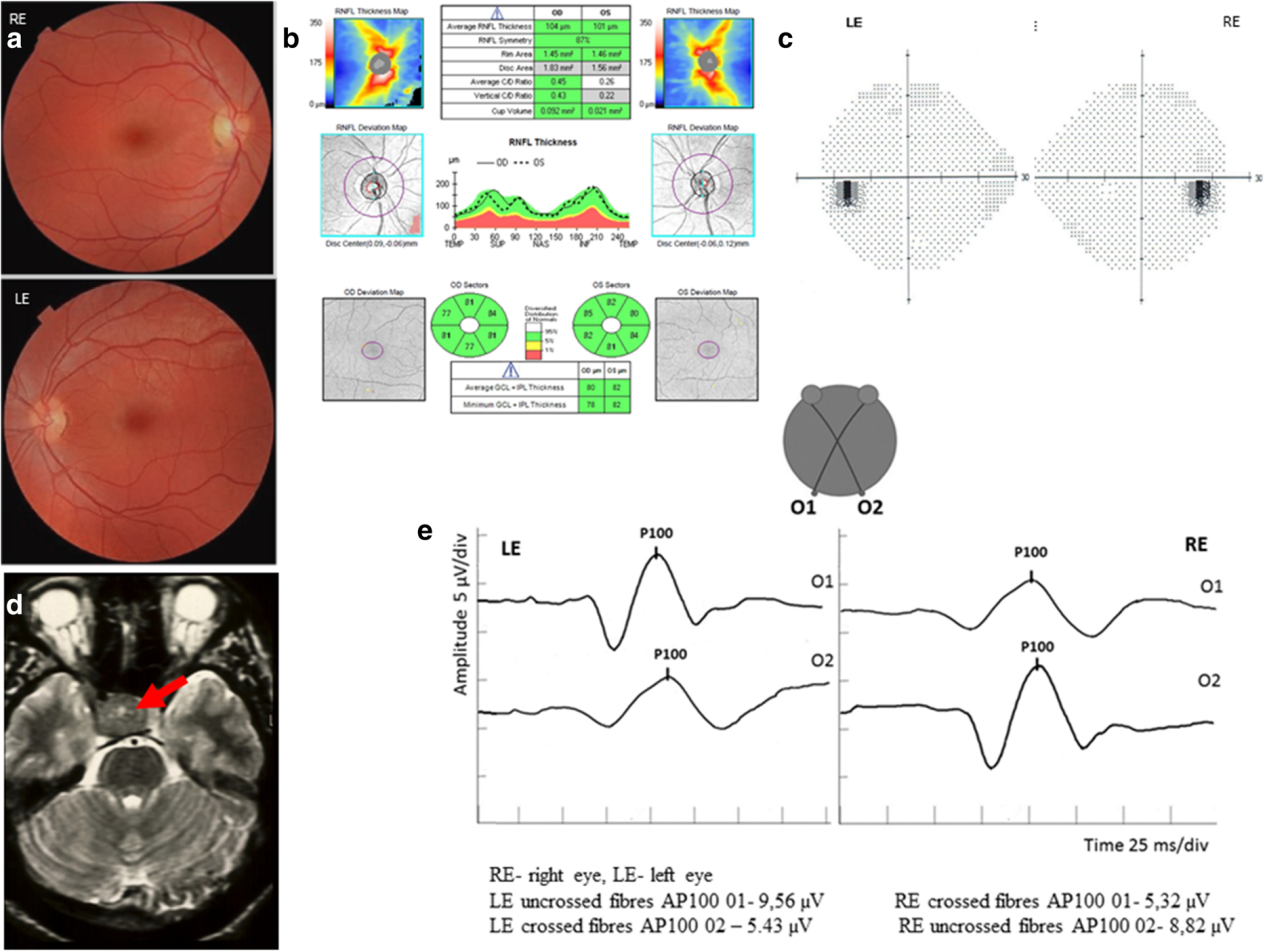
The normal results of fundus examination (**a**), OCT (**b**) and perimetry (**c**) in patient with microadenoma presents in MRI (**d**) and mVEP (1°4′) recording with amplitude of P100-wave reduction from a crossed fibres (the most prominent feature recording in our study)

The results of routine ophthalmological examination in both eyes of patients with microadenoma presence in MRI were as follows: DBCVA 1.0, intraocular pressure within the normal range (16.5 ± 1.89 mmHg), normal anterior and posterior segment of the eye and colour vision perception. Retinal sensitivity (MD 0.35 ± 0.99 dB, PSD 1.5 ± 0.49 dB) as well as circumpapillary RNFL (96.14 ± 7.8 µm) and GCC thickness (81.14 ± 4.36 µm) were within the normal range (Fig. 1).

### PVEP (1°4′)

**Fig. 2:**
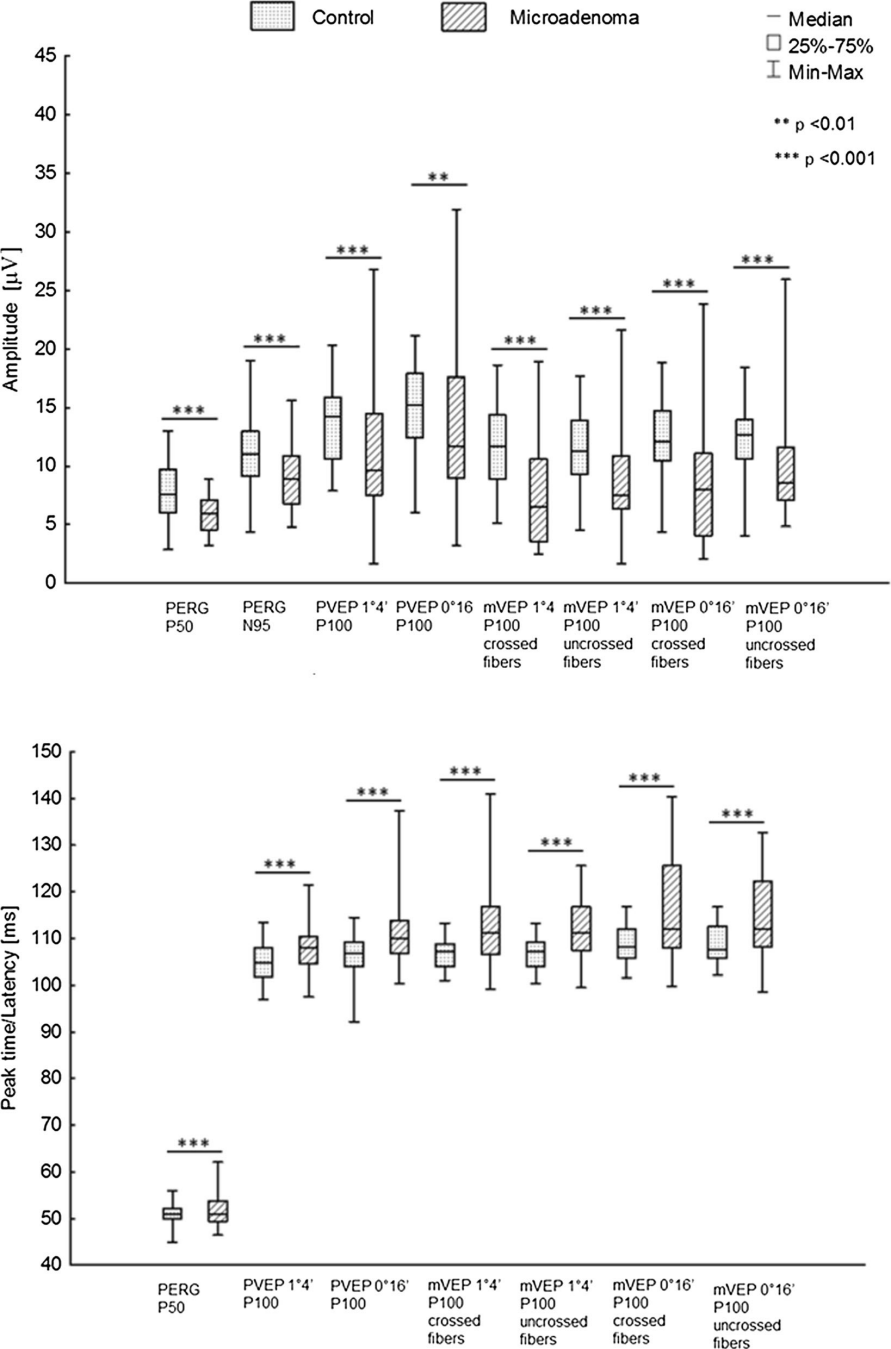
Comparison of the mean amplitude (**a**) and peak time/latency (**b**) of PERG/VEP of patients with microadenoma to controls (*n*-33 patients/*n*-66 eyes)

Statistically significant differences in AP100 and LP100 between patients with microadenoma and the healthy controls were detected (Fig. 2).

### PVEP (0°16′)

Statistically significant differences in AP100 and LP100 between the patients with microadenoma and the healthy controls were registered (Fig. 2).

In a group of patients with microadenoma, a statistically significant reduction of AP100 and increase of LP100 were obtained for both check sizes (*p* < 0.01).

When the eyes of the patients with microadenoma were analysed separately in comparison with normal range values, the PVEP results were as follows: check size (1°4′)—AP100 reduction (10/33 patients, 30.3%; 13/66 eyes, 19.7%), LP100 increase (6/33 patients, 18.18%; 7/66 eyes, 10.6%); check size (0°16′)—AP100 reduction (3/33 patients, 9.09%; 4/66 eyes, 6.06%), LP100 increase (7/33 patients, 21.21%; 11/66 eyes, 16.67%).

In PVEP, the most frequent abnormality was a AP100 reduction for large field size (1°4′).

**Fig. 3:**
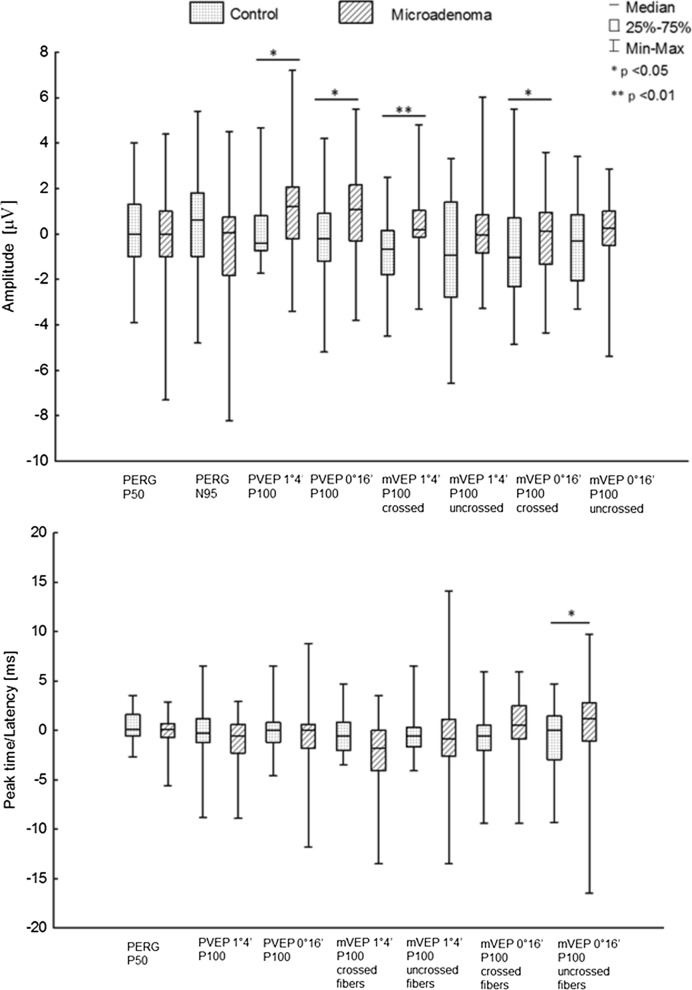
Comparison of mean interocular/interhemispheric asymmetry of amplitude and peak time/latency of PERG/VEP of patients with microadenoma to controls

The interocular asymmetry of PVEP is presented in Figure below (Fig. 3).

In group of patients with microadenoma, statistically significant interocular asymmetry of AP100 was observed for both check sizes (*p* < 0.05).

The sensitivity and specificity of the analysed parameters of PVEP are presented in Table 1.

**Table 1:** Analysis of the effectiveness of the diagnostic criterion of PERG/VEP

Parameter	PVEP				mVEP								PERG		
	P100-wave														
	1°4′		0°16′		1°4′				0°16′						
	A	L	A	L	Crossed		Uncrossed		Crossed		Uncrossed		P50-wave		N95-wave
					A	L	A	L	A	L	A	L	A	PT	A
Sensitivity	0.545	0.545	0.545	0.682	0.606	0.515	0.621	0.576	0.636	0.348	0.742	0.758	0.909	0.227	0.606
Specificity	0.864	0.742	0.803	0.697	0.938	0.938	0.854	0.917	0.813	1.00	0.729	0.375	0.470	0.955	0.742
Accuracy	0.701	0.671	0.635	0.721	0.777	0.747	0.712	0.764	0.755	0.708	0.731	0.694	0.741	0.558	0.735

*A* amplitude, *L* latency, *PT* peak time

Based on the AUC, the best way to diagnose patients with and without microadenoma has a LP100 of PVEP (0°16′). The cut-off point is 108.6, sensitivity 0.682 and specificity 0.697, AUC 0.721 (*p* < 0.001).

### mVEP (1°4′)

Statistically significant differences in AP100 and LP100 between the patients with microadenoma and the healthy controls were observed (Fig. 2).

### mVEP (0°16′)

Statistically significant differences in AP100 and LP100 between the patients with microadenoma and the healthy controls were found (Fig. 2).

In patients with microadenoma, mVEP (1°4′)/(0°16′) revealed a statistically significant AP100 reduction and an increase of LP100 from the crossed to the uncrossed fibres (*p* ≤ 0.001).

When the eyes of the patients with microadenoma were analysed separately in comparison with normal range value, the following abnormalities were observed in mVEPs: check size (1°4′)—an increase of the LP100 from the crossed (12/33 patients, 36.36%; 20/66 eyes, 30.3%) and the uncrossed fibres (13/33 patients, 39.39%; 21/66 eyes, 31.8%) and AP100 reduction from the crossed (13/33 patients, 39.39%; 23/66 eyes, 34.8%) and the uncrossed fibres (3/33 patients, 9.09%; 4/66 eyes, 6.1%); check size (0°16′)—an increase of the LP100 from the crossed (12/33 patients, 36.36%; 23/66 eyes, 34.8%) and the uncrossed fibres (13/33 patients, 39.39%; 23/66 eyes, 34.8%) and AP100 reduction from the crossed (11/33 patients, 33.33%;19/66 eyes, 28.8%) and the uncrossed fibres (6/33 patients, 18.18%; 7/66 eyes, 10.6%).

The most frequent changes registered in this test were the increase of the LP100 from the uncrossed fibres (1°4′) and theAP100 reduction of this wave from the crossed fibres (0°16′).

Additionally, a crossed asymmetry (the disturbances were maximal over the hemisphere contralateral to the stimulated eye) were registered as follows: check size (1°4′) AP100 (2/33 patients, 6.06.%; 2/66 eyes, 3.03%), LP100 (11/33 patients, 33.33%; 17/66 eyes, 25.76%); check size (0°16′) AP100 (3/33 patients, 9.09%; 4/66 eyes, 6.06%), LP100 (10/33 patients, 30.3%; 17/66 eyes, 25.76%).

The most frequently observed feature was the difference of LP100 of mVEP (1°4′) between the crossed and the uncrossed fibres.

The interhemispheric asymmetry of mVEP is presented in Figure above (Fig. 3).

In group of patients with microadenoma, statistically significant interhemispheric asymmetry of AP100 was observed for mVEP (1°4′)/(0°16′) from crossed fibres (*p* < 0.05).

Statistically significant interhemispheric asymmetry of latency was observed for P100 from uncrossed fibres of mVEP (0°16′) (*p* ≤ 0.033).

The sensitivity and specificity of the analysed parameters of mVEP are given in Table 1.

Based on the AUC, the best way to diagnose patients with and without microadenoma has the AP100 from the crossed fibres of mVEP (1°4′). The cut-off point is 7.595, sensitivity 0.606 and specificity 0.938, AUC 0.777 (*p* < 0.001).

### PERG

In the study group, statistically significant differences in AP50, AN95 and PTP50 were observed when compared to the control group (Fig. 2).

In group of patients with microadenoma, statistically significant AP50, AN95 reduction and increase of PTP50 were found (*p* ≤ 0.001).

When the eyes of patients with microadenoma were analysed separately in comparison with the normal range values, the PERG results were as follows: AP50 reduction (3/33 patients, 9.09%; 3/66 eyes, 4.55%), AN95 reduction (3/33 patients, 9.09%; 3/66 eyes, 4.55%), increase of PTP50 (7/33 patients, 21.21%; 11/66 eyes, 6.67%).

The most frequent abnormality in PERG was the increase of PTP50.

There was no statistically significant interocular asymmetry of PERG (Fig. 3).

The sensitivity and specificity of the analysed parameters in PERG are presented in Table 1.

Based on AUC, the best way to diagnose patients with and without microadenoma had AP50 as well as AN95 of PERG. The cut-off point is 9.34, sensitivity 0.606 and specificity 0.742, AUC 0.735 (*p* < 0.001).

Concurrent PERG/VEP abnormalities are presented in figure above (Figs. 2, 3).

A statistically significant amplitude reduction was observed in all analysed electrophysiological tests (*p* < 0.01).

A statistically significant increase of PTP50 was observed in PERG, and an increase of LP100 was registered in PVEP/mVEP (*p* ≤ 0.001).

A statistically significant interocular/interhemispheric asymmetry of amplitude was observed for P100 of PVEP (1°4′)/(0°16′) and mVEP (1°4′)/(0°16′) from crossed fibres (*p* < 0.05).

A statistically significant interhemispheric asymmetry of latency was observed for P100 from uncrossed fibres of mVEP (0°16′) (*p* ≤ 0.033).

**Fig. 4:**
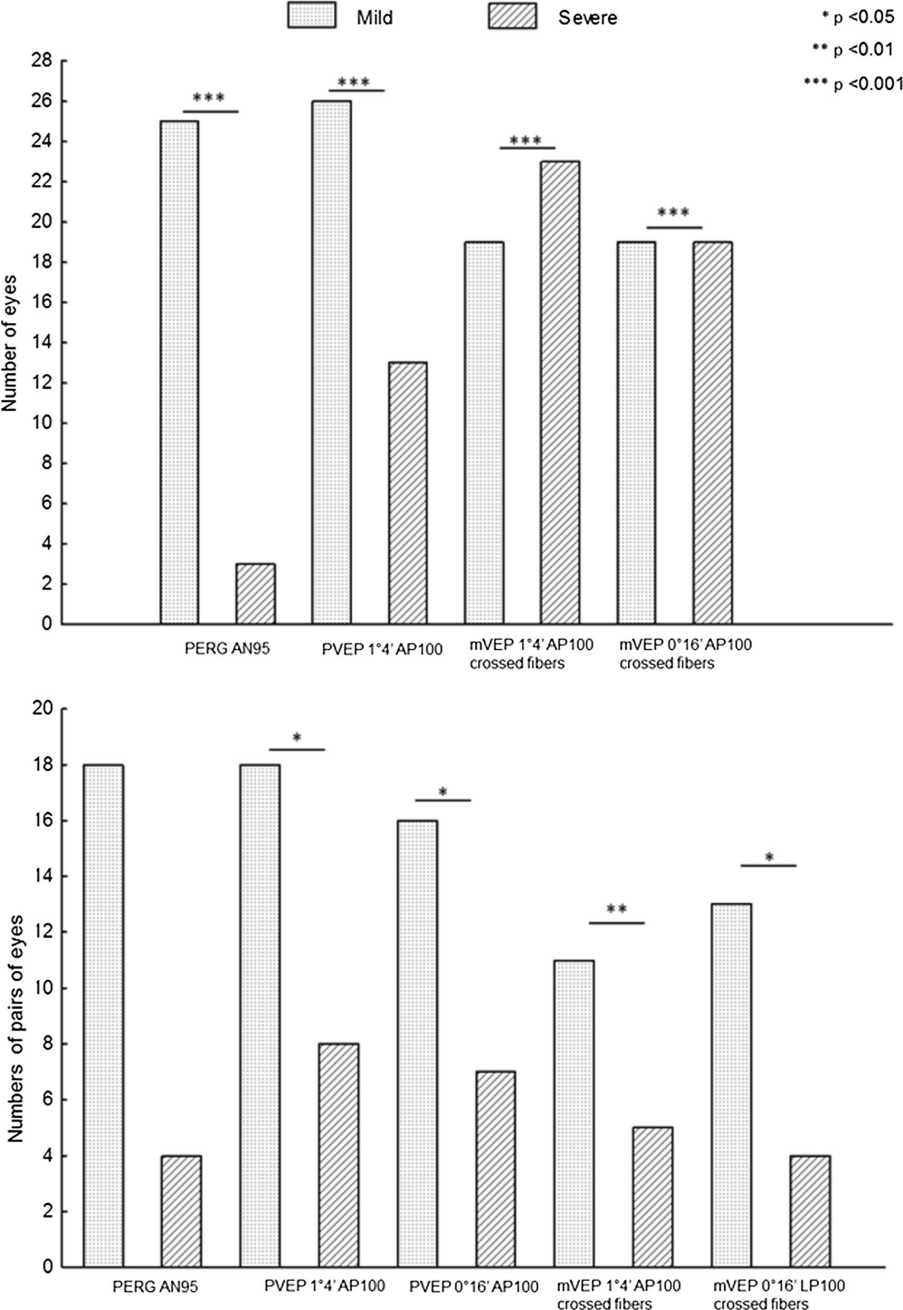
Histogram detailing the essential PERG/VEP findings in patients with microadenoma

The most significant selected abnormalities (values different from the norm ≥ 20%) occurred in the electrophysiological tests in patients with microadenoma which were classified as mild (± 1–2 SD) and severe (> ± 2SD) [23] (Fig. 4).

The most pronounced bioelectrical functional abnormality was observed in AP100 of mVEP (1°4′) from crossed fibres and in interocular AP100 asymmetry of PVEP (1°4′) (*p* < 0.05).

**Fig. 5:**
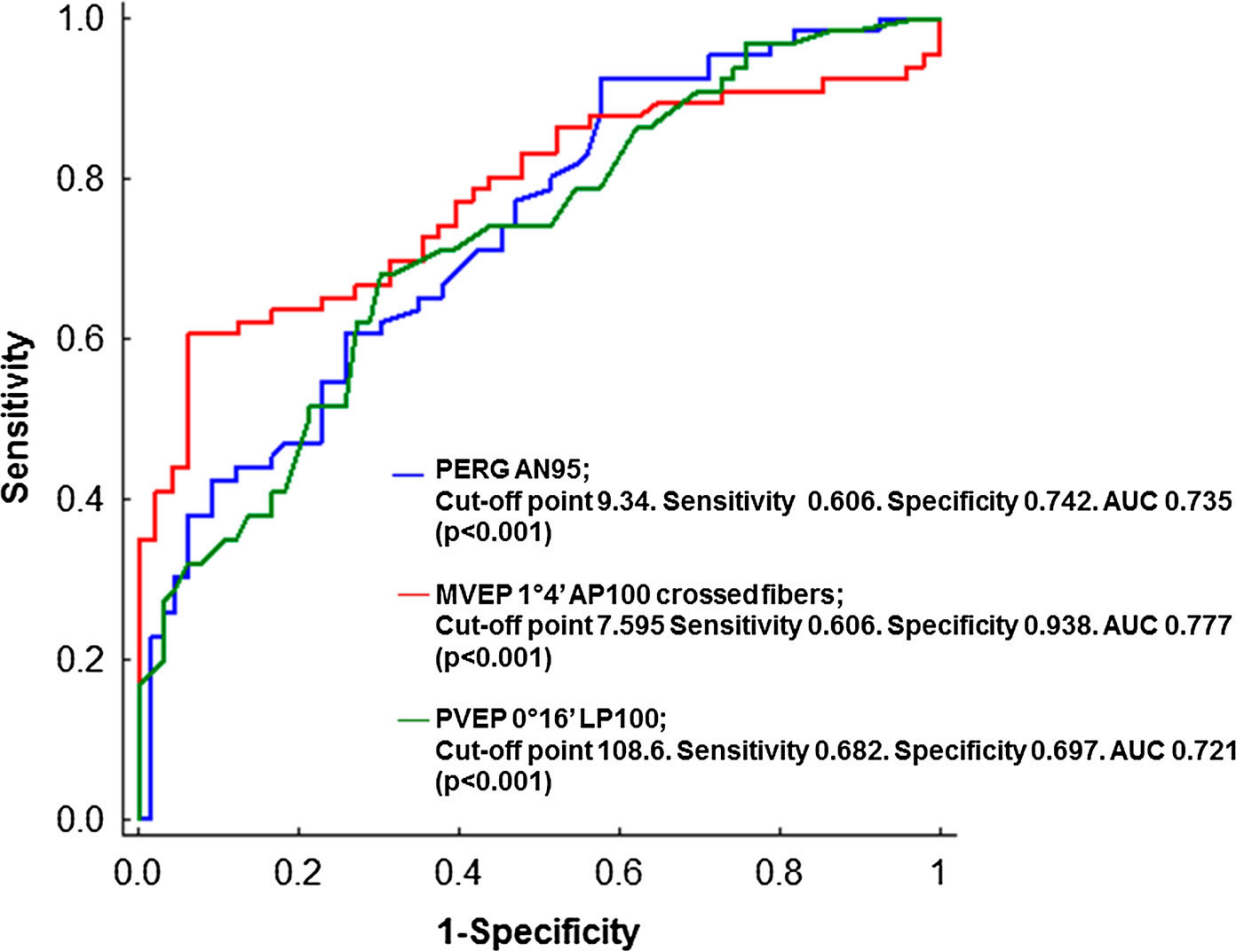
ROC curves—analysis of the effectiveness of diagnostic criterion of selected parameters of PERG/VEP

According to the data in the literature, AUC of 0.7–0.8 is considered acceptable and can diagnose patients with and without the disease or condition based on the test [24]. Although the value of all the parameters analysed in the study is comparable and acceptable in the diagnosis of visual pathway dysfunction in patients with microadenoma, the greatest diagnostic predictor is the AP100fromthecrossed fibres of mVEP (1°4′) (AUC0.777) (Fig. 5).

This parameter also indicates the downstream to the chiasm type of visual pathway dysfunction and its severity.

## Discussion

The results of the present study clearly demonstrate the influence of microadenoma on the electrophysiological function of the visual pathway. According to the algorithms, an ophthalmological consultation is recommended in patients with pituitary tumour [25, 26]. The electrophysiological tests were performed in patients with microadenoma even without chiasmal compression in MRI, ophthalmic symptoms, and changes in other ophthalmological examinations, because the data known from the literature mentioned in this article and our experience suggest that, in patients with microadenoma, a dysfunction of the visual pathway may be present. Although the tests are not routinely used in diagnosis, they provide useful information about prognosis of visual deficits and the location of the lesion [6, 10, 19, 20, 27].

According to our best knowledge, this is the first research which describes many aspects (VEP, PERG) of the optic nerve and the ganglion cells dysfunction in the group of patients with microadenoma.

The PVEP is a reliable method for assessing the function of the optic chiasm in patients with a pituitary tumour [6–8, 20–22]. In our study, even with microadenoma, the PVEP changes were detected. The most frequent abnormality was the P100 wave amplitude reduction after stimulation with large check sizes (1°4′). The best ability to diagnose patients with microadenoma had a P100 wave latency of PVEP (0°16′) with a sensitivity of 54.5% and the specificity of 86.4% (AUC0.721) (Table 1).

Another electrophysiological test which can be useful in diagnosis of visual pathway dysfunction in patients with pituitary tumour is the mVEP [9, 15, 18, 19, 28]. It was noted that the most frequent feature was the increase of the P100 wave latency (1°4′) from uncrossed fibres, but from crossed fibres a reduction in the amplitude of this wave (0°16′) was observed. The latency of P100wave increase indicates on conduction disturbances and may be a subclinical feature of the optic nerve dysfunction. According to data from the literature, the P100 wave amplitude reduction confirms the RGCs axons dysfunction and/or structural damage [29]. Based on the mentioned mVEP changes, it is sensible to conclude that the intensity of dysfunction was more prominent in the crossed fibres.

An additional feature which was detected in our study and strongly suggested a chiasmal lesion was a crossed asymmetry, which does not occur in normal individuals and manifested as the difference between the crossed and the uncrossed fibres of P100 latency of mVEP (1°4′) [19].

In mVEP, the best ability to diagnose the patients with microadenoma had an amplitude of P100 wave of mVEP (1°4′) from the crossed fibres with a sensitivity of 60.6% and a specificity of 93.8% (AUC 0.777) (Table 1).

The PERG can be useful in the diagnosis of patients with pituitary tumours [10, 28, 29]. In our study, the most frequent abnormality was the increase of P50-wave peak time, which indicated not only RGC impairment but may also suggest a cone dysfunction in the macular region [30]. However, the best ability to diagnose patients with microadenoma in PERG had an amplitude of N95-wave with a sensitivity of 60.6% and a specificity of 74.2% (AUC 0.735) (Table 1).

Based on our findings, we can conclude that all the analysed electrophysiological tests have the comparable and acceptable ability to differentiate between patients with microadenoma and healthy subjects because the value of AUC of most of the parameters was more than 0.7 [24]. However, these study results revealed a slight superiority of the AP100 of mVEP (1°4′) from the crossed fibres (Table 1, Fig. 5).

Similar abnormalities in the electrophysiological tests were also observed by other authors mentioned in this article, but mostly in patients with macroadenomas. The weaknesses of the previous studies included the small numbers of cases, poorly defined groups (different diseases in chiasmal region, different type of pituitary adenoma or stage of the disease), only one test or measurement parameters applied, and methodology not always consistent with ISCEV standards. Therefore, it is not possible to compare previous results with our findings. Only one study described the electrophysiological changes in patients with microadenoma using VEP [9]. In this study abnormal mVEP was obtained in four patients (4/13; 31%), but was associated with the visual field defect in static perimetry. The P100 wave amplitude reduction and/or interhemispheric and interocular asymmetry of P100 wave latency was noted. No changes in PVEP were observed.

The exact mechanism responsible for visual pathway dysfunction produced by microadenoma is unclear. One of the possible explanations is swelling of the pituitary gland [31]. The sellaturcica is not an expansible cavity. The previous study results suggested that even a small increase of pituitary tumour size of a fraction of millimetre may be the cause of intrasellar hypertension which leads to visual pathway dysfunction [32–34]. The ischemia of the optic chiasm produced by microadenoma was probably responsible for the obtained visual pathway dysfunction detected in the electrophysiological test.

The anatomical relationship between the optic chiasm and the pituitary gland makes the optic pathway susceptible to influence by lesions expanding from the sellaturcica. As it is known, the nasal retinal fibres from the retina are crossed in the chiasmal and temporal retinal fibres continue the way uncrossed 53/47%, respectively [35], but there might be also variation in the percentage of these fibres. The chiasm derives its blood supply from the inferior and superior anastomotic group of vessels. There is evidence that the body of the chiasm receives its blood supply only from the inferior anastomotic group of vessels, while the lateral parts of the chiasm are supplied from both the inferior and the superior groups [36]. It seems rational to expect that the increase of intrasellar pressure will be more pronounced in the group of the optic nerve fibre layers obtaining its blood supply from one source only. This may explain why intrasellar hypertension is mainly manifested in the crossed fibres. The early consequence of the ischemia of the chiasm is the disruption of neural conduction along the axons, impairment of anterograde and retrograde axoplasmic flow finally leading to demyelination. Long-lasting chiasmal compression can increase the loss of nerve fibres and the retrograde degeneration of RGCs [37, 38].

Other possible causes of visual pathway abnormalities are changes in metabolites, trophic factors or proteases associated with the development of neoplasm in the immediate microenvironment [10, 11, 39].

The results of presented study may have a clinical value and show the significance of electrophysiological tests in the early diagnosis of patients with microadenoma because it can be registered even without other signs of the optic pathway pathology. The observations from literature and our findings indicate that the role of the electrophysiological tests seems greater at individual patient level in order to establish whether a particular individual has mild or severe dysfunction along the visual pathway. Additionally, the results of electrophysiological tests can suggest that the type of dysfunction is either downstream to the chiasm when the changes are observed in VEP or upstream when PERG is altered, which would suggest possible retrograde RGC dysfunction [23].

The weakness of this study is the lack of follow-up with patients with visual pathway dysfunction. So, we cannot prove but only suggest that patients with microadenoma are at risk of optic nerve atrophy in the future.

In conclusion, the visual pathway dysfunction in patients with microadenoma can be detected by the electrophysiological tests like mVEP, PVEP, PERG.

It is sensible to conclude that the diagnosis of visual pathway dysfunction at the early stage of the pituitary tumour may be a reason for introducing or changing the pharmacological treatment and, in some cases, deciding on neurosurgical treatment.

We recommend that the electrophysiological tests should be included in the algorithm of management of patients with microadenoma.

## Data Availability

The datasets generated and/or analysed during the current study are available from the corresponding author on reasonable request.

## Declarations

### Statement on the welfare of animals

This chapter does not contain any studies with animals performed by any of the authors.
